# Gaining Clarity on the Claritɣ Algorithm

**DOI:** 10.1007/s12028-023-01797-z

**Published:** 2023-07-31

**Authors:** Josef Parvizi, Kapil Gururangan, Dan Knickerbocker, Baharan Kamousi, Raymond Woo

**Affiliations:** 1 Department of Neurology & Neurological Sciences, Stanford University School of Medicine, 300 Pasteur Drive A343, Stanford, CA 94305 USA; 2 Research and Development Division, Ceribell Inc., Sunnyvale, CA USA; 3 Department of Neurology, David Geffen School of Medicine, University of California Los Angeles, Los Angeles, CA USA

We read the report by Dr. Villamar and his colleagues [1] with great interest, and we congratulate the authors on presenting a well-balanced evaluation of Ceribell’s Claritɣ seizure burden algorithm [2]. We do fully agree with the authors’ conclusion that artificial intelligence is not ready to replace neurologists. As some of the developers of the Claritɣ algorithm, we also add that our algorithm was never designed to replace trained clinicians who interpret a patient’s electroencephalogram (EEG) in the context of the patient’s clinical presentation.

Here, we hope to clarify what the Claritɣ algorithm was designed to do and, more importantly, what it was not designed to do. Claritɣ was designed to detect seizures and calculate the load of such activity (i.e., “seizure burden,” which we defined as the cumulative percentage of 10-s epochs with seizures across all channels over the prior 5-min period). An alarm is generated at the bedside when the seizure burden exceeds 90% (i.e., when 4.5 out of 5 min contain seizure activity, indicative of impending status epilepticus). The output of the algorithm is the load of seizure activity and not the labeling of each seizure incident. Although the detection of brief seizures is important for diagnostic purposes, brain injury occurs only with a high seizure burden [3]. Because, by design, point-of-care EEG is meant to guide treatment decisions at the bedside, Claritɣ was designed to alert only for high seizure burden. As such, the Claritɣ algorithm should not be mistaken for a seizure or spike detection tool designed to detect the presence of any epileptiform activity.

In an ideal world, trained EEG specialists (i.e., neurologists with board certification in clinical neurophysiology or epilepsy) would be available within minutes 24/7 to accommodate the interpretation of urgent EEG studies. However, even well-resourced centers with on-call, EEG-trained neurologists experience gaps and delays in obtaining and interpreting EEG data, especially during after-hours [4], leaving intensivists and emergency physicians at the bedside to evaluate patients at risk for nonconvulsive status epilepticus empirically and solely based on clinical suspicion without real-time objective data. Point-of-care EEG systems have emerged as one solution to this palpable problem. In this regard, it is important to acknowledge the hundreds of cases at Dr. Villamar’s institution where the algorithm had performed correctly. Furthermore, exceptional cases presented in the study by Villamar et al. [1] should not be freely extrapolated to all patients in need of point-of-care EEG monitoring. While the authors highlight a few cases in which Claritɣ missed seizure activity, they also point out that the performance of this algorithm was satisfactory in the majority of EEGs performed at their institution.

In certain patient populations (including survivors of cardiac arrest), electrographic seizure detection will be an especially challenging task for EEG-trained neurologists and machine learning algorithms alike due to high rates of extracerebral signals (e.g., myogenic activity, shivering, or artifacts from surrounding critical care apparatus, etc.) and subtle seizures due to decreased amplitude and fluctuation along the ictal-interictal continuum [5]. Fortunately, a much larger population of patients undergoing EEG monitoring may not be subject to the same complexities.

**Fig. 1:**
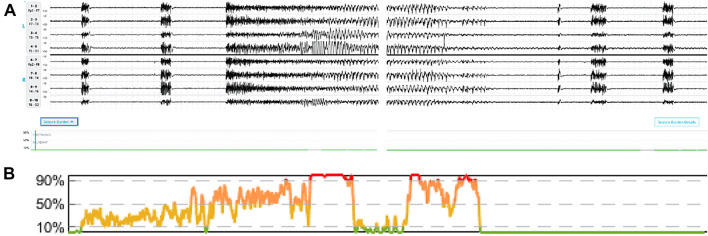
Performance of updated Claritɣ algorithm v6.0. **a** Two-minute segment of ictal EEG from patient 4 from Villamar et al. [1] with original seizure burden trend showing 0% seizure burden throughout the recording (displayed as a green horizontal line in trend bar below EEG montage). The patient had fragmented seizures. EEG display settings: ± 50 μV sensitivity, 1 Hz high-pass filter, 30 Hz low-pass filter, 60 Hz notch filter. **b** Updated seizure burden trend generated by Claritɣ algorithm version 6.0 demonstrating more accurate detection of higher seizure burden, displayed as an increase in the percentage of 10-s epochs detected to have seizure activity within a rolling 5-min window (*y*-axis) from 0–10% (green) to 90–100% (red) over time (*x*-axis). EEG, electroencephalogram (Color figure online)

As with many technologies in medicine, there are opportunities for improvement. Our team, similar to many others who have developed algorithms for clinical use, regularly works with users to retrain the algorithm to improve accuracy for missed cases in version upgrades approximately twice a year. Since the initial cases reported by Villamar et al. [1], two new versions of Claritɣ have been released with clear improvements. The latest release (version 6.0 released November 2022) already shows improvement regarding two of the cases reported in this study (Fig. 1). We expect future releases to demonstrate even greater improvement.

In closing, we welcome healthy skepticism and further research into the accuracy of our algorithm. With continuous dialogue between clinicians and corporate engineers, machine learning algorithms will continue to improve over time as more cases are aggregated to train and retrain classifiers. Our neurology and neurocritical care community should recognize that point-of-care EEG tools with improved algorithms will only empower them rather than replacing them. To broaden access to prompt EEG monitoring for critically ill patients across the entire country and to reduce disparities of care, new technologies ought to be adopted and optimized through honest feedback (such as the one led by Dr. Villamar and his team) rather than being viewed with pure incredulity.
